# Impact of Real-Time Comorbidity Capture on Clinical Escalation and Harm Prevention in Hospitalized Patients: A Benchmarking and Statistical Gap Analysis

**DOI:** 10.7759/cureus.95758

**Published:** 2025-10-30

**Authors:** Samy Allam, Christine Gharib

**Affiliations:** 1 Quality and Health Data Integrity, Arrowhead Regional Medical Center, Colton, USA; 2 Medical Education, California University of Science and Medicine, Colton, USA

**Keywords:** comorbidity documentation, informatics, patient safety, real-time ehr, sirs

## Abstract

Background

Systemic documentation deficiencies in hospitalized patients with high-acuity conditions such as systemic inflammatory response syndrome (SIRS) can compromise clinical decision-making, risk adjustment, and patient safety. Despite well-established evidence linking chronic conditions like chronic kidney disease (CKD), diabetes mellitus (DM), and liver failure to adverse outcomes, these comorbidities are often underrepresented in electronic health records (EHRs). This study introduces a statistically rigorous, informatics-assisted framework to benchmark and quantify real-world documentation gaps, advancing the field of clinical documentation integrity (CDI) through actionable metrics.

Objective

The objectives of this study are to evaluate the accuracy of comorbidity documentation in adult inpatients with SIRS and acute organ dysfunction by applying a benchmarking model based on peer-reviewed prevalence data and to assess the clinical, operational, and financial implications of these documentation deficits.

Methods

In this retrospective observational study, 82 adult patients admitted with a principal diagnosis of SIRS (ICD-10-CM R65.11) were analyzed using structured chart abstraction from EPIC EHR data. Six high-impact comorbidities were evaluated. Observed documentation rates were compared against literature-derived expected prevalence benchmarks. Gap scores were calculated as proportional differences, and one-sample z-tests were used to assess statistical significance, stratified by age and sex.

Results

Five of six comorbidities demonstrated statistically significant underdocumentation, with liver failure (expected: 57%, observed: 6.1%; *p* < 0.001), DM (53% vs. 18.3%; *p* < 0.001), and HTN (78% vs. 36.6%; *p* < 0.001) showing the most severe deficiencies. Gap scores ranged from 0.39 to 0.89, revealing systemic failure in documenting clinically relevant conditions. Age and sex stratification further exposed disparities in documentation behavior. Notably, underdocumentation did not correlate with inpatient mortality (*p* = 0.89), indicating systemic gaps unrelated to clinical outcome visibility.

Conclusion

This study provides a validated, reproducible framework for identifying and quantifying clinical documentation gaps in real-time. The comorbidity capture gap model integrates seamlessly with EHRs and aligns with CMS risk adjustment frameworks such as Hierarchical Condition Category (HCC) and Diagnosis-Related Group (DRG). Its application can enhance clinical accuracy, improve institutional quality metrics, and inform scalable, AI-augmented CDI interventions. The findings underscore a critical opportunity to shift from reactive to proactive documentation practices, transforming the reliability of health data for risk stratification, harm prevention, and healthcare performance reporting.

## Introduction

Real-life documented realities may not reflect patients' stories in hospital admissions, especially when recording real-time comorbidities and effects of impending conditions. Patients admitted to the hospital often suffer from multiple comorbidities, including hypertension, diabetes, heart disease, malnutrition, and frailty. These conditions can complicate the patient’s hospital experience, extend their length of stay (LOS), and increase their risk of clinical deterioration and mortality. For instance, diabetic patients have an increased risk of delayed wound healing, infection, and poor glycemic control, all of which can prolong their hospital course and lead to additional patient complications [1]. Other variables, such as age, body habitus, and their correlation with documented comorbidities, are thoroughly discussed in many studies, including the one by Allard et al. This study indicated that age-adjusted admission rates for patients with all-cause hospitalizations were 631.3 and 454.8 per 1,000 patients, and the mean length of stay was 8.2 and 7.1 days, respectively, for those with and without diabetes. Similarly, malnutrition exacerbates patients' risk of infection, poor wound healing, and reduced immune function, thereby worsening overall health prognosis [2]. Malnutrition is prevalent in hospital settings, anywhere from 15-70%. Patient malnutrition has been shown to increase LOS and healthcare costs. Caccialanza et al. demonstrated that patients at nutritional risk had a LOS median of 13 days compared to those not at risk, at a median of seven days [3]. Another key comorbidity is frailty, which causes diminished physiological reserves and a subsequent increase in the risk for patient infections, falls, and additional adverse events [4]. Frail patients have a significantly higher risk of hospital-treated infections compared to non-frail patients. This risk rises with the severity of frailty, with frail patients having up to 1.78 times increased risk of hospital-treated infections compared to non-frail patients. In a hospital setting, frail patients comprise a disproportionate share of hospital-acquired infections, particularly patients having three or more frailty deficits [4,5]. Patient comorbidities can complicate health management and often require individualized care plans that incorporate these risks and adjust the intensity and care provided to decrease the chance of complications. Managing such complexities requires an interdisciplinary approach that accounts for all relevant comorbid conditions.

The interplay between several comorbidities can exacerbate patient deterioration and create many complex challenges for healthcare providers. This includes physical or mental illnesses, or both, where guidelines do not define borderline illnesses. For instance, patients with end-stage renal disease (ESRD) experience compounded risks when combined with other conditions, such as heart failure and diabetes. Diabetic patients with ESRD and cardiovascular disease are reported to have higher rates of all-cause mortality and recurrent hospitalizations compared to patients without comorbidity [6]. The risk of developing ESRD is higher in heart failure patients with diabetes, and once ESRD develops, the one-year mortality rate is high at 32% [7]. These comorbidities increase the patient's likelihood of adverse clinical outcomes, relating to prolonged hospitalizations, fluid overload, or adverse cardiovascular events. When considering these risks, it is further heightened alongside conditions such as hypertension or coronary artery disease comorbidity with ESRD, increasing vulnerability to acute kidney injury (AKI) and requiring specialized care to monitor patient status and acute events carefully [8]. Thus, this necessitates that hospitalized patients with multiple comorbidities receive highly coordinated care that addresses all interconnected health risks. Multidisciplinary healthcare teams that utilize specialists in all relevant fields, such as cardiology, nephrology, and nutrition, play an essential role in managing the complexity of these patients. The KDIGO 2025 guidelines acknowledge that lifestyle modifications (such as blood pressure control, dietary sodium restriction, maintaining a healthy weight, and increasing physical activity) may be critical in slowing disease progression and improving overall health in autosomal dominant polycystic kidney disease (ADPKD) patients [9]. However, the guidelines highlight a critical gap: the lack of robust clinical trials specifically studying the impact of structured lifestyle interventions on hard clinical outcomes such as eGFR decline, cyst growth, or quality of life measures post-hospitalization. Similarly, psychosocial issues, including depression, anxiety, and coping with chronic pain and uncertainty about disease progression, are common but understudied in ADPKD populations. There is no standardized approach to providing mental health support to ADPKD patients, and few randomized controlled trials have evaluated interventions like counseling, support groups, or cognitive-behavioral therapy explicitly tailored to this population. As a result, clinicians have limited evidence-based tools to address these significant aspects of patient well-being. Not adequately addressing these needs can result in worsened patient health outcomes, subsequent prolonged hospitalizations, and avoidable complications, highlighting the importance of preventive and comprehensive patient assessment and care.

Preventable harm

Timely and accurately documenting patient comorbidities is vital in ensuring the appropriate level of care is provided. Failure to do so may result in missed clinical opportunities for early intervention. The Surviving Sepsis Campaign emphasizes the importance of early recognition and timely intervention in sepsis management, which is critically dependent on accurate and prompt clinical documentation. Early signs of comorbidities, such as chronic kidney disease, diabetes, or immunosuppression, significantly influence both the risk of sepsis progression and the patient’s response to treatment. Documentation that captures these comorbidities at the point of care supports rapid risk stratification, appropriate antibiotic selection, and escalation of care, aligning with the campaign’s hour-1 bundle recommendations. Furthermore, robust documentation enhances the clinical validity of severity scoring systems and predictive tools that rely on comorbidity indexing. Failure to document these conditions in real time may delay sepsis recognition, inappropriate therapeutic interventions, and potential deterioration in patient outcomes [10]. Therefore, integrating structured documentation practices into sepsis protocols fulfills regulatory and coding standards and supports evidence-based clinical decision-making.

Litchford et al. highlighted other conditions that insufficient capture, documentation, and reporting may affect, such as malnutrition [11]. Patients who are malnourished but do not have clinical documentation in their medical chart may not be referred to a nutritionist or receive adequate nutritional interventions to prevent complications relating to wound infection or delayed recovery time [12]. Studies report up to 66% of patients are at risk of malnutrition, and 84% are (pre-)frail [13]. About half of these patients may suffer from both malnutrition and frailty, highlighting the need for joint assessment. In particular, malnutrition is often under-documented and not recognized in hospital settings, which prevents targeted nutritional support that would increase the patient’s chances of recovery [14]. Frail patients also often require more intensive and consistent monitoring, as less invasive interventions have a higher risk of complications. A lack of early documentation may subject patients to unnecessary treatments that increase clinical harm events, such as falls, pressure injuries, or delirium [15].

Early documentation also plays a significant clinical role in guiding the medical team’s decision-making for determining the appropriate level of patient care. Suppose a patient’s risk for pressure injuries is not documented. In that case, the care team is more likely to overlook essential and preventative measures, such as repositioning schedules or utilizing specialized mattresses [16]. Lack of these interventions will put the patient at a higher risk of developing pressure injuries, resulting in prolonged hospital stays and increased healthcare costs. The incomplete or inaccurate documentation of comorbidities may also delay the necessary implementation of critical care protocols, such as starting early warning systems or receiving specialist consultations, which can aid in mitigating the various risks associated with comorbidities [17]. Subsequently, patients may suffer preventable harm and poor clinical outcomes, underlining the importance of accurate and comprehensive clinical documentation as part of a high-quality care delivery model.

Reactive documentation

Traditional clinical documentation practices are often reactive, meaning that key clinical risks may not be appropriately identified until after the onset of adverse effects, such as infections or cardiovascular events [18]. In many healthcare settings, patient documentation of comorbidities is typically completed after a patient’s condition has deteriorated; thus, the opportunity for early intervention generally has passed [19]. A patient at risk for malnutrition may not be referred for dietary consultation until their nutritional status has worsened, which typically leads to further complications. The delayed clinical recognition, documentation, and response to patient risk factors highlight the limitations of traditional documentation workflows, which usually capture essential data only after an adverse clinical event. These typical workflows can result in missed opportunities for early risk prevention and harm reduction, increasing the likelihood of adverse events, and potentially prolonging patient hospital stay and costs [20].

Reactive documentation can lead to fragmented communication between healthcare providers on the patient care team. Suppose a patient is at risk for developing pressure injuries without early documentation. In that case, nursing staff may not implement necessary preventative measures, such as using specialized support surfaces or ensuring the patient has been moved every few hours [21]. The lack of comorbidity documentation on the patient’s medical record fails to alert essential healthcare team members to the patient’s need for specialized interventions, ultimately delaying medical care. Lacking timely and comprehensive documentation poses a significant barrier to providing patients with proactive care. Utilizing a proactive and comprehensive documentation system supported by advanced health informatics is essential for improving clinical workflow and bypassing preventable patient harm [22]. Accurate physician chart documentation can facilitate appropriate patient care decisions, reduce medical errors, and promote health system planning and resource allocation to support quality patient care, especially in patient comorbidity. 

Timely documentation

Clinical escalation protocols, including early warning systems, specialist consults, and safety bundles, are critical for identifying and responding to deteriorating patients. These protocols rely on accurate and timely documentation of patient high-risk comorbidities. Early warning systems utilize various clinical and physiological data to identify patients at risk of deterioration. For instance, the escalation planning process in hospice inpatient units is reported as effective, with 94% of patients having an escalation plan documented, and 88% of the escalation events matching the plan [23]. Escalation protocols have shown overall positive results for patient clinical outcomes. Plans formed in advance that are well documented in combination with appropriate clinical judgment have demonstrated positive outcomes in individual situations to ensure that proper clinical treatments are given to produce positive patient outcomes. Without comprehensive patient documentation of comorbidities such as frailty, malnutrition, or ESRD, these systems may fail to recognize the full extent of patient risk [24]. Accurate and early documentation is essential for these systems to trigger an appropriate response to improve clinical outcomes, such as activating rapid response teams, referring specialists, or initiating interventions necessary for patient stabilization [25]. This kind of system requires prompt recognition and management of critical care events. Specialist consults, which concern patient comorbidity, may trigger, but will likely not be initiated promptly and proactively if these conditions are not adequately documented, leading to treatment delays and worsened patient outcomes.

Safety bundles are evidence-based collections of designed practices clinically used to prevent specific patient complications, such as infections. This method heavily relies on the timely identification of comorbidities. Safety bundles may differ widely between practices; however, they are typically organized into domains such as readiness, recognition, prevention, response, reporting, and system learning [26]. For instance, if patient malnutrition is not documented promptly, safety measures created to prevent infections may be overlooked or not implemented. Safety bundles should thus be adapted to meet the resources available at each facility, and standardization within an institution should also be encouraged. Accurate charting documentation is essential for ensuring that all patient care team members have access to the information necessary to make clinically informed decisions regarding escalation protocols [24-26]. Without this information, there is an increased risk of fragmented care, leading to compromised patient care and safety due to avoidable adverse outcomes.

Advanced informatics tools

Real-time detection and flagging of comorbidities through advanced informatics tools have emerged as an essential component of patient safety infrastructure. These tools can be integrated into electronic health records (EHRs) to automatically flag high-risk patient comorbidities as soon as they are documented [27]. This flagging system provides clinicians with alerts to initiate appropriate patient interventions. EHR systems can use predictive algorithms to detect patients at risk for complications based on their specific history to trigger preventative actions [28]. Informatics tools in EHR systems can infer logical comorbidity networks through associations consistent with current medical understanding. Vulnerable patients are thus detected early in their hospitalization to prompt more frequent assessments and develop targeted interventions that prevent further deterioration. Comorbidity networks can therefore assist doctors in diagnosing patients faster and minimizing potential missed diagnoses. Using real-time alerts is critical in hospitals where timely and early interventions can significantly impact patient outcomes to reduce the likelihood of avoidable complications [29].

These detection tools enhance clinical decision-making by providing real-time access to patient data that highlights their comorbidities and associated risks. By incorporating real-time detection systems into the clinical workflow, healthcare providers can make faster and more informed decisions that improve overall patient safety and quality of care [30]. Detection and characterization of comorbidity is an invaluable aid and prominent challenge within healthcare. Detection tools support efficient detection and provide evidence-based insights into comorbidity progression. Advanced informatics tools also support a more proactive approach to patient care by enabling healthcare teams to address key risks before they result in clinical deterioration. Predictive analytics can identify patients at risk for AKI or sepsis, for instance, based on their medical history, comorbidities, and real-time clinical data. This allows for earlier clinical intervention and improved efficient management for clinical decision support [31]. As healthcare systems rely on digital tools and technology, real-time detection and flagging of patient comorbidities will become integral to harm prevention and improving patient safety. The consequences of diagnostic errors have been insufficiently studied, and the interaction of multiple comorbidities or borderline diagnoses is often underreported. Healthcare reimbursement system constraints frequently limit the time for thorough assessments, prompting physicians to order unnecessary tests. This practice strains financial resources, as these patients often fall outside conventional diagnostic frameworks, resulting in costly evaluations with limited effectiveness. In this study, we will discuss five different interventions involving newly introduced data analytics and machine learning tools to implement harm prevention strategies in patients' EHRs based on documented data, aiming to address care gaps concurrently and improve hospital outcomes for these patients. This study aims to assess the documentation gap of key comorbidities in patients with SIRS and acute organ dysfunction using real-time benchmarking and informatics-assisted data capture. The hypothesis follows that high-impact comorbidities, such as chronic kidney disease, heart failure, and diabetes, are underdocumented in patients who clinically meet criteria for these diagnoses, resulting in a gap between expected and observed prevalence in EHR data.

## Materials and methods

Data sources and population

EHR data were extracted for all adult inpatients meeting suspected sepsis criteria between January 2023 and July 2025 admitted to Arrowhead Regional Medical Center (ARMC). Arrowhead Regional Medical Center is a community-based Acute care Hospital in Colton, California. Patients admitted to this hospital often maintain multiple comorbidities and Social Determinants of Health. The selection of this population is intended to analyze and demonstrate the scientific rigor and importance of robust documentation practices and their impact on disease progression. This study utilized a retrospective observational design to assess the accuracy of comorbidity documentation in patients diagnosed with SIRS of non-infectious origin, accompanied by acute organ dysfunction. Patients were included if they had ≥2 SIRS criteria with a suspected infection diagnosis. The patient cohort was identified with the ICD-10-CM diagnosis code R65.11. All patients were required to have a principal diagnosis of SIRS, with additional criteria ensuring clinical evidence of acute organ dysfunction. Patients initially documented under SIRS criteria but later redacted were excluded to ensure accurate inclusion criteria, and only index admissions were included in the final dataset.

**Figure 1:**
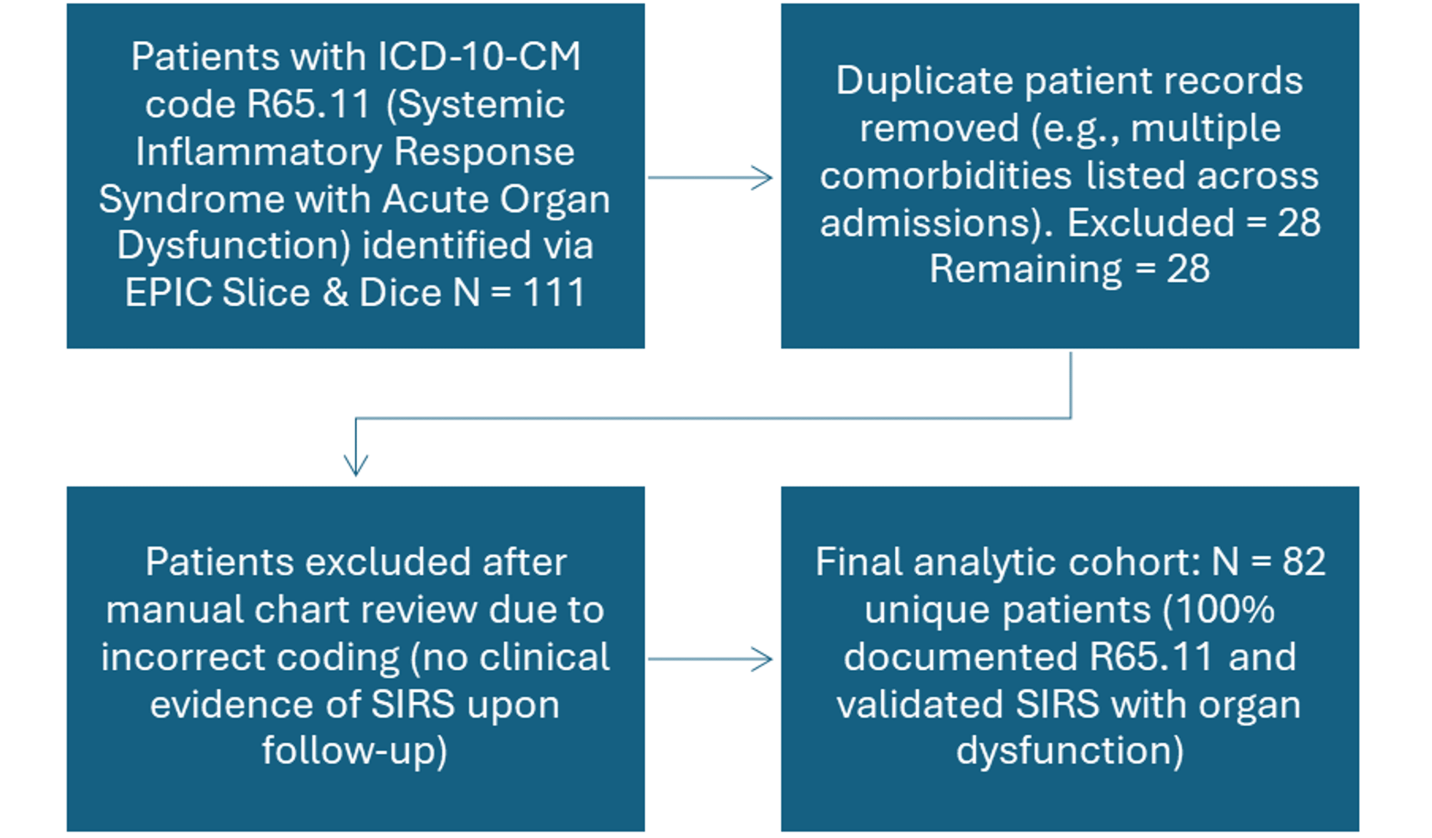
STROBE-Compliant Flow Diagram SIRS: Systemic inflammatory response syndrome; STROBE: STrengthening the Reporting of OBservational studies in Epidemiology

In this analysis, according to Figure 1, 111 patients were identified with a principal SIRS diagnosis, as determined by ICD-10 code R65.11. This cohort was initially retrieved from the institutional EPIC electronic health record system. Upon detailed review, duplicate records from patients with multiple documented comorbidities were consolidated to ensure unique patient representation. One patient was also excluded due to reclassification following clinical reassessment, as their condition was no longer consistent with SIRS criteria as initially suspected at admission. Consequently, the final analytical cohort consisted of 82 patients. The demographic composition of the studied cohort included 57 male patients (69.5%) and 25 female patients (30.5%) stratified across four age categories: nine patients (10.9%) < 30 years old, 24 patients (29.4%) 30-50 years old, 31 patients (37.8%) 51-70 years old, and 18 patients (21.9%) >70 years old. The prevalence of the following expected high-impact comorbidities was documented: CKD in eight patients (9.8%), DM in 15 patients (18.3%), HTN in 30 patients (36.6%), anemia in 23 patients (28.0%), liver failure in five patients (6.1%), and cardiovascular disease in 17 patients (20.7%). During hospitalization or shortly thereafter, 17 patients (20.7%) were documented as expired.

This cohort was used for a comprehensive data analysis of key comorbidities documented during patient admission, stratified by demographic values. The emphasis was on identifying gaps in clinical documentation pertinent to quality and reimbursement metrics.

Table 1 shows age distribution and reveals a broad representation across adult age groups, with the largest subgroup being patients aged 51-70 years (37.8%), followed by those aged 30-50 years (29.3%). Notably, 22% of the cohort was older than 70, highlighting the inclusion of a high-risk geriatric population, which is often more vulnerable to adverse outcomes due to comorbidities and frailty. Regarding sex distribution, males constituted a significant majority at 69.5%, suggesting a potential sex-related skew in either SIRS incidence, documentation, or admission patterns at the study site. The in-hospital mortality rate of 20.7% reflects a substantial burden of disease severity within this group. It underscores the clinical importance of early and accurate documentation of comorbidities for this high-risk population. As seen in later tables, these demographic patterns set the stage for stratified analysis, allowing for further exploration into whether age or sex contributed to variations in documentation practices. Table 1 provides essential context for interpreting documentation trends and underscores the importance of tailored clinical documentation integrity (CDI) strategies that consider demographic risk factors.

**Table 1:** Cohort Demographics Demographic characteristics of the 82-patient cohort hospitalized with SIRS and acute organ dysfunction, including distribution by age group, sex, and in-hospital mortality. SIRS: Systemic inflammatory response syndrome

Variable	N (%)
Age Group	
<30	9 (11.0%)
30–50	24 (29.3%)
51–70	31 (37.8%)
>70	18 (22.0%)
Sex	
Male	57 (69.5%)
Female	25 (30.5%)
Mortality	
Died	17 (20.7%)
Survived	65 (79.3%)

Objective and hypothesis

The primary objective of this study is to evaluate the accuracy and completeness of clinical documentation for high-impact comorbidities in patients diagnosed with SIRS and acute organ dysfunction. Specifically, the study aims to determine whether certain chronic conditions indicating organ dysfunction, such as CKD, diabetes mellitus (DM), hypertension (HTN), anemia, liver failure, and cardiovascular disease (CVD), are being consistently captured in the EHR at the time of patient admission.

To achieve this, we employed a real-time, informatics-assisted chart review and benchmarking approach. We compared the observed documentation rates in the hospital’s EHR system to nationwide well-established prevalence benchmarks derived from peer-reviewed literature and national databases. This methodology allowed us to quantify the documentation gap for each comorbidity, expressed as a “gap score,” and to assess statistical significance using inferential testing.

We hypothesize that several high-impact comorbidities, particularly CKD, DM, and heart failure, are significantly underdocumented in this patient population relative to their expected prevalence. Such underdocumentation may not only obscure the true clinical complexity of the patient but may also delay escalation of care, affect risk adjustment metrics, and compromise both quality reporting and reimbursement. By identifying these gaps, the study seeks to inform targeted CDI strategies that enhance patient safety, support timely clinical decision-making, and improve institutional performance on key quality measures.

Retrospective data signals were reviewed to identify the presence or absence of the following intervention

This study reviewed retrospective EHR data to determine whether structured documentation of high-impact comorbidities was present during hospital admission for patients diagnosed with SIRS and acute organ dysfunction. The focus was on identifying whether comorbid conditions, such as CKD, diabetes, HTN, anemia, liver failure, and CVD, were explicitly captured in structured fields, problem lists, or coded diagnoses upon admission. These comorbidities were flagged as “Yes” only if documentation existed in a standardized, retrievable form that could inform clinical decision-making or contribute to severity indexing. Absence of such structured entries was considered a missed documentation opportunity, regardless of whether the condition was later implied in narrative notes or progress updates.

This approach identified potential gaps between clinical presentation and formal documentation, critical in risk stratification, treatment planning, and compliance with quality-based reimbursement programs. By anchoring the analysis on structured documentation at the point of admission, the study highlights a key intervention point where accurate and complete data capture can influence downstream patient outcomes and institutional performance metrics. Reviewing retrospective data signals allowed the research team to systematically compare real-world documentation practices against evidence-based expectations, offering insights into where and why underdocumentation may occur within existing workflows.

Data collection and variable definitions

Clinical data were manually extracted from the EPIC EMR system at ARMC and entered into a structured Microsoft Excel database. For each patient, the following variables were collected: patient ID, age, sex, ethnicity, and a predefined set of comorbidities relevant to managing SIRS and related outcomes.

The comorbidities tracked included CKD, DM, HTN, anemia, congestive heart failure (CHF), and liver failure. Each condition was coded as “Yes” if explicitly documented in the patient’s chart or problem list, and “No” if there was no documentation. Mortality status (alive or deceased) and other clinically significant findings were recorded. To ensure completeness, an additional chart review was conducted to identify complex or multifactorial conditions that may not have been coded but were relevant to the patient’s clinical course and SIRS-related risk profile.

Expected comorbidities and benchmarking

A comprehensive review of peer-reviewed literature and national quality benchmarking data was conducted to determine the high-priority comorbidities likely to be present within the SIRS patient population. Sources included public health repositories such as the Agency for Healthcare Research and Quality (AHRQ), the National Center for Biotechnology Information (NCBI), OpenICPSR, and clinical studies indexed in PubMed. The goal was to establish expected prevalence benchmarks for comorbidities frequently associated with SIRS or sepsis-like inflammatory syndromes.

CKD was assigned an expected prevalence of 18%, based on population-level studies analyzing systemic inflammation markers and renal disease using large-scale health databases [32]. The expected prevalence of DM was set at 53%, informed by retrospective chart reviews of diabetic status in septic ICU patients, incorporating both clinical documentation and laboratory findings [33]. HTN was benchmarked at 78%, as reported in prospective epidemiological studies profiling sepsis patients' demographics and clinical features in tertiary care settings [34].

Anemia prevalence was estimated at 66%, supported by data from prospective cohort studies examining anemia trends, hemoglobin levels, and transfusion needs among critically ill patients [35]. Liver failure was benchmarked at 57%, derived from a clinical cohort study assessing the incidence of hepatic dysfunction in the context of systemic inflammatory response [36]. Finally, CVD prevalence was set at 34%, extrapolated from a multicenter observational study that examined the relationship between SIRS and cardiovascular complications in patients admitted to cardiac intensive care units [37].

These literature-derived benchmarks, as shown in Table 2, served as expected prevalence rates for comorbidities with high clinical relevance but often inconsistent or incomplete documentation in electronic medical records. They were used as reference values to calculate capture gap scores, a metric quantifying the difference between expected comorbidity prevalence and the actual documentation observed in the study cohort. This approach identified potential underdocumentation or missed opportunities for diagnostic clarity and coding precision.

**Table 2:** Comorbidity and Respective Prevalence Expected prevalence of key comorbidities derived from peer-reviewed literature and population-level datasets used to benchmark documentation gaps.

Comorbidity	Prevalence
Chronic Kidney Disease	18%
Diabetes Mellitus	53%
Hypertension	78%
Anemia	66%
Liver Failure	57%
Cardiovascular Disease (proxy: CHF)	34%

Table 2 summarizes the expected prevalence rates of key comorbidities in patients with SIRS, based on literature benchmarks and national health data. HTN (78%) and DM (53%) emerged as the most common expected conditions, consistent with known associations between cardiometabolic disease and systemic inflammation. Anemia (66%) and liver failure (57%) also demonstrated high expected prevalence, reflecting the common occurrence of multisystem dysfunction in critically ill populations. CKD, while less prevalent at 18%, remains clinically significant due to its strong correlation with poor outcomes in inflammatory states. Cardiovascular disease, estimated at 34% using congestive heart failure (CHF) as a proxy, highlights the interplay between SIRS and cardiac complications. These benchmarked rates serve as reference standards to identify potential under-documentation within the study cohort, offering insight into gaps in clinical documentation that could impact risk stratification, severity adjustment, and reimbursement accuracy.

Analytical approach

A comorbidity capture gap analysis was conducted to evaluate the completeness and accuracy of comorbidity documentation among patients with SIRS. This analytical method compares observed documentation patterns within the study cohort to expected prevalence benchmarks derived from peer-reviewed literature and large-scale population datasets, as shown in Table 3. The goal was to identify discrepancies that may reflect underdocumentation of clinically significant conditions, affecting severity indexing, quality metrics, and reimbursement accuracy.

**Table 3:** Comparison of Observed vs. Expected Prevalence Observed documentation rates compared to expected prevalence for six high-impact comorbidities, including confidence intervals, z-scores, and statistical significance.

Comorbidity	Expected Prevalence	Observed Prevalence	95% CI (Observed)	Z-Score	P-Value
CKD	0.180	0.098	[0.034, 0.162]	-1.93	0.053
Diabetes	0.530	0.180	[0.097, 0.263]	-6.35	< 0.001
Hypertension (HTN)	0.780	0.370	[0.266, 0.475]	-8.96	< 0.001
Anemia	0.660	0.280	[0.183, 0.377]	-7.26	< 0.001
Liver Failure	0.570	0.061	[0.009, 0.113]	-9.31	< 0.001
Cardiovascular Disease	0.340	0.210	[0.122, 0.298]	-2.49	0.013

The observed prevalence of each comorbidity was calculated by dividing the number of patients with the comorbidity documented in the EMR by the total number of patients in the cohort, then multiplying by 100 to express the result as a percentage:

Observed Prevalence (%) = (Number of Patients with Documented Comorbidity ÷ Total Number of Patients) × 100

This metric provides a real-world snapshot of how frequently specific conditions are recognized and recorded within the EMR system. It reflects provider documentation habits and serves as the empirical basis for assessing documentation performance.

A gap score was computed for each comorbidity to quantify the degree of under-documentation. This score was calculated using the formula:

Gap Score (%) = [(Expected Prevalence − Observed Prevalence) ÷ Expected Prevalence] × 100

These formulas are adapted from standard proportional difference calculations commonly used in epidemiologic benchmarking and healthcare quality measurement studies to compare observed versus expected rates [38-44]. The gap score provides a normalized measure of documentation discrepancy by expressing the shortfall as a proportion of what is expected based on literature benchmarks. A higher gap score indicates a larger deviation from expected norms and may signal clinical or coding oversight. For example, if diabetes is scheduled for 53% of patients but documented in only 30%, the gap score of 43.4% highlights a significant under-capture.

A documentation capture rate threshold of 30% was applied to flag clinically meaningful deficiencies. Comorbidities with observed prevalence below 30% of their expected benchmark were identified as having potentially significant documentation gaps. This threshold helps prioritize areas for CDI intervention, particularly when under-capture could distort case mix index, severity of illness scoring, or risk-adjusted outcomes.

To determine whether the observed documentation rates differed significantly from expected values, one-sample proportion z-tests were conducted for each comorbidity. These tests assess whether the difference between the observed prevalence and the literature-derived benchmark is statistically significant or likely due to chance. A two-tailed level of significance of < 0.05 was established, with p-values of less than 0.05 indicating a statistically significant result. Additionally, 95% confidence intervals (CIs) were calculated around the observed prevalence values to measure the precision of the estimates and account for sampling variability.

Together, this analytical framework provides a robust and interpretable method for identifying gaps in clinical documentation. It highlights areas where improved diagnostic capture and more accurate charting could enhance the fidelity of administrative data, better reflect patient complexity, and inform targeted Clinical Documentation Integrity CDI strategies.

Table 3 presents a comparative analysis of expected versus observed prevalence for six key comorbidities in the SIRS study cohort. Each comorbidity’s documentation rate in the EMR was statistically tested against benchmarked expectations using one-sample z-tests, with 95% confidence intervals provided to assess estimate precision. CKD had an expected prevalence of 18%, but was observed in only 9.8% of patients. Although this under-documentation trend is notable, the z-score of -1.93 and a p-value of 0.053 indicate that the difference was marginally non-significant at the 0.05 threshold. This suggests a possible under-capture that warrants attention but did not meet conventional criteria for statistical significance. DM was expected in 53% of patients, but was only documented in 18%. The wide discrepancy produced a z-score of -6.35 and a p-value < 0.001, confirming a statistically significant and clinically meaningful gap in documentation. The 95% confidence interval for the observed prevalence [0.097, 0.263] further supports the reliability of this finding. HTN showed the most significant documentation gap among all conditions evaluated. While the expected prevalence was 78%, only 37% of patients had documented hypertension. This yielded a z-score of -8.96 and a p-value < 0.001, indicating strong statistical significance and reinforcing the need for improved capture of this prevalent comorbidity. Anemia also demonstrated significant under-documentation, with an expected rate of 66% but an observed prevalence of only 28%-the z-score of -7.26 and p-value < 0.001 support a statistically robust finding. The confidence interval [0.183, 0.377] confirms that the observed rate falls below benchmark expectations. Liver failure had one of the lowest observed documentation rates at 6.1%, despite an expected prevalence of 57%. This substantial gap produced a z-score of -9.31 and a highly significant p-value (< 0.001), suggesting that liver failure may be severely under-recognized or under-coded in the EMR data. CVD (using CHF as a proxy) had an expected prevalence of 34%, while documentation reflected only 21%. The z-score of -2.49 and p-value of 0.013 indicate a statistically significant under-documentation, albeit less severe than the gaps observed for HTN, liver failure, or anemia. Five of the six comorbidities demonstrated statistically significant under-documentation compared to literature-based prevalence benchmarks. These findings underscore critical opportunities for improving clinical documentation practices, particularly in high-impact areas like HTN, diabetes, and liver failure, where under-capture may affect risk adjustment, severity scoring, and reimbursement.

Statistical rationale and data visualization

Descriptive statistical analysis was employed due to its relevance in quality improvement research and practical use in spreadsheet-based platforms. Calculations were performed using Microsoft Excel, which allowed for the integration of conditional formatting to flag comorbidities with low visual capture rates. This facilitated intuitive data exploration and rapid identification of documentation deficiencies. Inferential statistics were not applied in this phase of the analysis, as the primary objective was not to determine causality or measure effect size, but rather to generate actionable, system-level insights into existing clinical documentation practices that could inform targeted improvement strategies.

Bar graphs were developed to visually compare the observed documentation rates of each comorbidity against their corresponding expected prevalence benchmarks derived from the literature. Each chart included two plotted series-observed prevalence and expected prevalence-allowing for a side-by-side comparison that clearly highlighted areas of under-documentation. This visual format enabled easy identification of target areas for CDI and was a communicative tool for engaging stakeholders in quality initiatives. All data analyses and visualizations complied with institutional privacy standards and were conducted under an IRB exemption for retrospective chart review.

Analytical framework and data representation

We developed a quantitative comorbidity capture gap analysis framework to identify critical documentation gaps among high-impact comorbidities in patients with SIRS. This methodology benchmarks expected versus observed prevalence rates of specific chronic conditions within a clinically homogenous patient cohort. In contrast to traditional documentation improvement strategies, which often rely on retrospective audits, provider query logs, or qualitative chart reviews, our approach applies a real-time informatics model designed to detect discrepancies between clinically indicated conditions and those formally documented in the EMR.

The framework integrates structured ICD-10-CM coding data with condition-specific clinical indicators derived from patient problem lists, laboratory values, medication administration records, and encounter diagnoses. Expected prevalence rates were estimated using a combination of published literature benchmarks and internal institutional data from comparable SIRS populations. We generated capture gap scores for each targeted comorbidity by comparing these benchmarks to actual documentation patterns. These scores provide a quantitative measure of documentation performance, not merely by recording frequency, but by clinical plausibility, highlighting cases where the absence of documentation may represent a significant omission rather than random variation.

A key strength of this model lies in its applicability for real-time deployment. The logic behind the capture gap framework can be embedded into clinical decision support systems (CDSS) or sepsis early warning tools to alert providers or CDI specialists when a patient’s recorded comorbidities do not align with the expected clinical complexity suggested by their presentation. This directly impacts coding accuracy, reimbursement, and risk-adjusted quality reporting.

Moreover, the model aligns with risk adjustment methodologies used by the Centers for Medicare & Medicaid Services (CMS), including the Hierarchical Condition Category (HCC) model and Diagnosis-Related Group (DRG) classifications. Both frameworks depend on accurately documenting chronic conditions to reflect patient complexity and predict healthcare resource utilization. By identifying and addressing under-documented comorbidities, our approach enhances the accuracy of severity indexing, risk scoring, and institutional benchmarking. Ultimately, this framework provides a scalable, data-driven method to improve documentation practices, optimize reimbursement, and accurately represent clinical acuity across quality and performance metrics.

## Results

A total of 82 unique patients with a principal diagnosis of SIRS of non-infectious origin (ICD-10-CM: R65.11) were included in the final analysis. The cohort was predominantly male, with 57 patients (69.5%) identified as male and 25 (30.5%) as female. The age distribution was as follows: nine patients (11.0%) were under 30 years old, 24 patients (29.3%) were between 30 and 50 years old, 31 patients (37.8%) were aged 51 to 70 years, and 18 patients (21.9%) were aged >70 years old.

Beyond documentation quality, this figure has broader implications for hospital operations and financial performance. Accurate recording of comorbidities is pivotal in risk adjustment methodologies used in models such as CMS-HCC and DRGs. Underdocumented conditions can lead to underestimating patient severity, missed reimbursement opportunities, and distorted quality metrics. Thus, this chart reflects documentation behavior and indirectly informs risk modeling, coding accuracy, and institutional benchmarking efforts.

**Figure 2:**
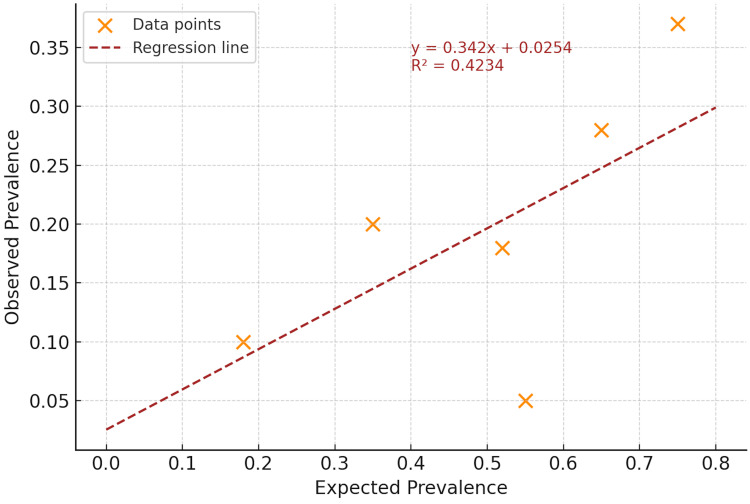
Linear Regression of Expected and Observed Prevalence

Throughout the study, there was a dire need to implement an application dashboard that can be a key foundation for running reports, especially at All Patient Refined Diagnoses Related Groups APR-DRGs, especially when quantifying the severity of illness SOI and Risk of Mortality ROM profiles. The scatterplot in Figure 2, titled “Expected Prevalence and Observed Prevalence,” visualizes the relationship between benchmarked (expected) and actual (observed) documentation rates of key comorbidities in the SIRS cohort. Each orange dot represents a specific comorbidity, plotted by its expected prevalence on the x-axis and its observed prevalence on the y-axis. The dotted trendline indicates a positive linear relationship (y = 0.342x + 0.0254), with an R² value of 0.4234, suggesting a moderate correlation between expected and observed values and revealing significant variability.

Visualizing outliers, interpretation, and relevance

The data points deviating furthest below the regression line represent outliers with substantial documentation gaps. These conditions are expected to occur at relatively high rates but are documented less frequently in practice. Most notably, liver failure and diabetes appear to fall well below the line, implying severe under-documentation relative to their expected prevalence. Liver failure, with an expected prevalence of around 57% but observed at nearly 6%, likely accounts for the lowest data point on the vertical axis. Diabetes, expected at 53% but documented in only 18%, also stands out as a significant outlier.

Conversely, comorbidities closer to the regression line, such as cardiovascular disease and chronic kidney disease, exhibit better alignment between expectation and documentation, though they still reflect under-capture. The highest point near the top right likely represents HTN, which, although underdocumented, is captured more consistently compared to other conditions.

Figure 2 provides a robust quantitative and visual method for identifying outliers in clinical documentation. The slope of the trendline (< 1) and modest R² value reinforce that observed documentation consistently lags behind clinical expectations. The outlier points call attention to specific conditions where documentation efforts are not keeping pace with the clinical burden, making this visualization a valuable tool for CDI teams, coders, and informatics leaders seeking targeted improvement.

Quantifying gaps via an assigned scoring system

In this study, gap scores were derived through a structured comorbidity capture gap analysis framework that quantitatively compared the expected prevalence of key chronic conditions, sourced from peer-reviewed literature and population-level epidemiological data, with their observed documentation rates in the EMR. Each gap score was calculated using the formula: (Expected Prevalence − Observed Prevalence) ÷ Expected Prevalence, yielding a standardized metric that reflects the proportion of clinically likely cases that went undocumented. This approach differs significantly from prior studies that rely on retrospective coding audits, provider query rates, or subjective chart reviews, which often lack consistency, real-time applicability, and benchmarking power. We ensured methodological rigor and reproducibility by grounding our model in clinical informatics, integrating structured data fields (ICD-10-CM codes, problem lists, lab results, medication orders), and using literature-based prevalence benchmarks. The resulting gap scores provide a meaningful, scalable way to detect under-recognition of high-impact comorbidities in real-world inpatient data. Importantly, this method quantifies documentation deficiencies and highlights missed opportunities for accurate risk adjustment, reimbursement optimization, and quality metric performance. It offers a robust foundation for CDI, health IT integration, and administrative decision-making.

**Figure 3:**
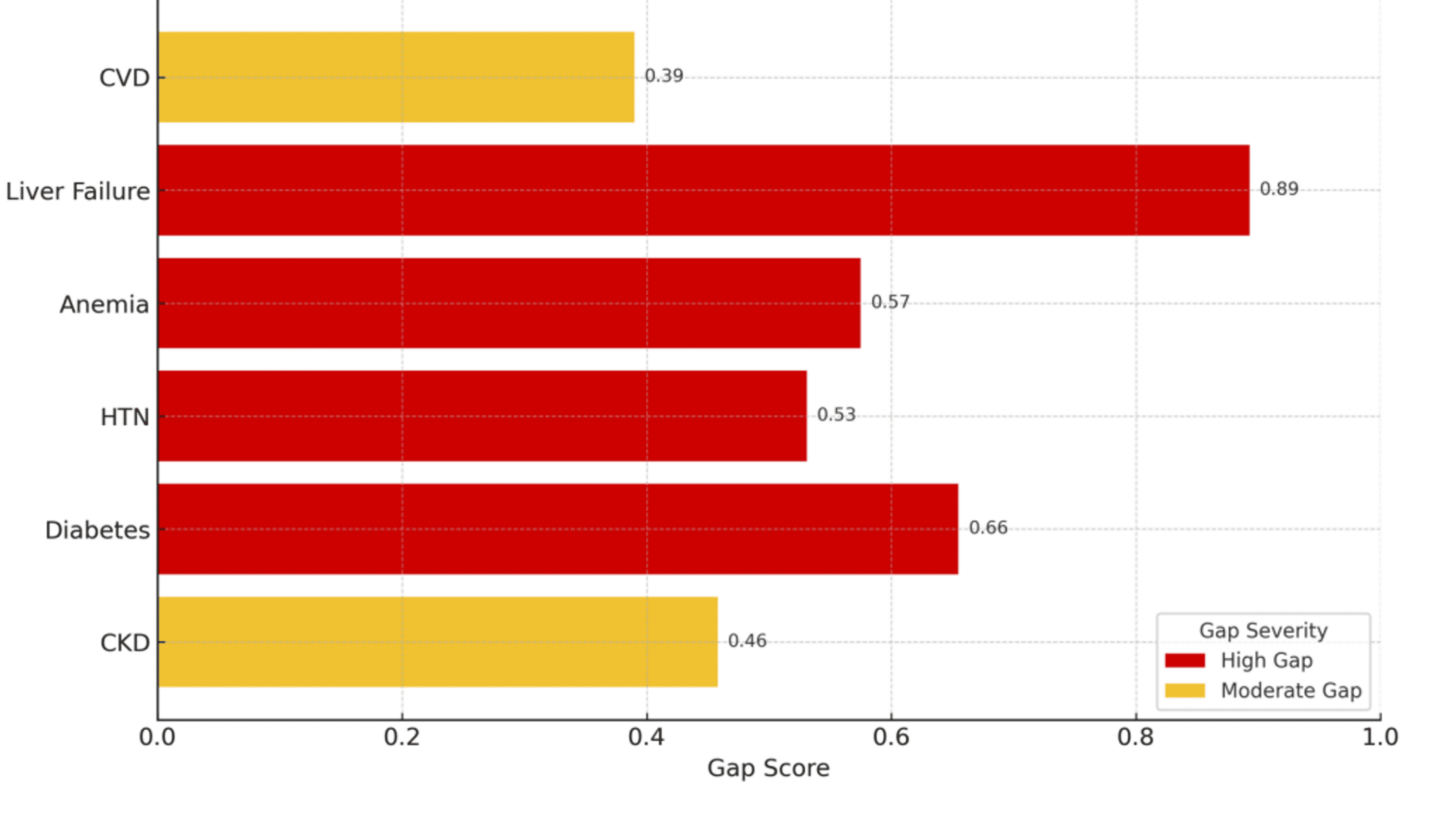
Comorbidity Documentation Gap Scores Bar chart ranking comorbidities by documentation gap score; higher values reflect greater underdocumentation severity CVD: Cardiovascular disease; HTN: hypertension; CKD: chronic kidney disease

Figure 3, titled “Comorbidity Documentation Gap Scores,” highlights critical disparities between clinically expected and actually documented chronic conditions in patients with SIRS. The horizontal bars quantify the gap score for each comorbidity, which is defined as the proportion of expected cases that lack proper documentation in the EMR. Notably, liver failure demonstrates the most severe deficiency, with a gap score of 0.89, indicating that nearly 90% of expected cases were undocumented. Diabetes, anemia, and HTN also show high gap scores (ranging from 0.53 to 0.66), underscoring pervasive under-recognition of these impactful comorbidities despite their direct clinical relevance. The color-coded severity scale distinguishes between high (red) and moderate (yellow) documentation gaps, emphasizing that only two conditions, CVD and CKD, fell into the moderate range, though still reflective of significant under-capture.

Figure 3 is a stark reminder of the lack of documentation intensity in inpatient settings, particularly for conditions integral to risk adjustment models, case mix indexing, and mortality profiling. The visual impact of these gaps points to systemic issues in how comorbidities are captured, either due to insufficient provider documentation, inadequate coding processes, or missed opportunities for clinical query. Bridging these gaps is not merely an administrative task but a quality imperative. Robust documentation practices reflect patient complexity and ensure alignment with reimbursement frameworks such as CMS-HCC and DRG. Thus, this visualization calls for immediate and targeted gap closure strategies-including real-time clinical decision support, enhanced CDI workflows, and provider education- to strengthen inpatient care documentation's accuracy, equity, and financial sustainability.

Age-related variations in clinical documentation

Our findings reveal a transparent age-related gradient, with a steep increase in documented comorbidities from younger to older age groups, which is evident. Patients under 30 had the fewest documented comorbidities (n ≈ 6), mostly consisting of HTN and anemia, with minimal or absent documentation of conditions like CVD and liver failure. The 30-50 age group shows a modest rise in documentation, but a dramatic escalation occurs in the 51-70 and >70 age categories. These older groups account for the highest volume of documented comorbidities, approaching 35-38 total, dominated by hypertension, anemia, diabetes, and an increasing representation of cardiovascular and liver disease. This pattern aligns with epidemiological expectations, as chronic disease burden increases with age. However, the clinical relevance lies in what is documented and what may still be under-documented. For instance, while anemia and HTN are consistently recorded in older groups, CKD and liver failure remain relatively infrequent, even in patients over 70, despite their known age-related prevalence. This disparity suggests that although documentation volume increases with age, certain high-risk conditions may still be under-recognized due to a lack of clinical prompts or Clinical Documentation Integrity CDI support.

**Figure 4:**
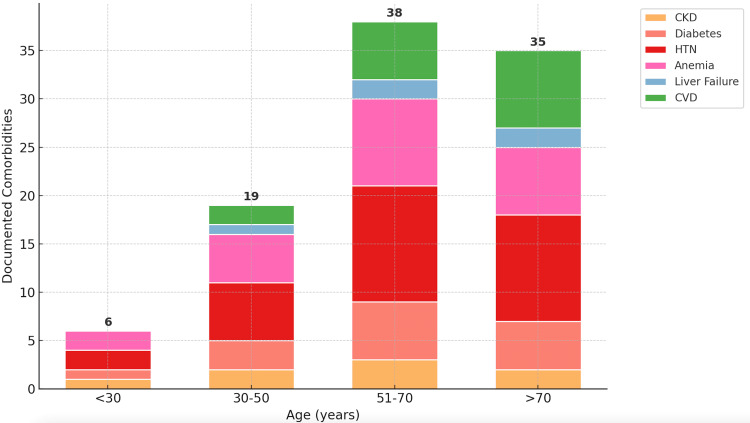
Documented Comorbidities by Age Group Distribution of documented comorbidities by age categories provides insight into age-related variation in documentation. CVD: Cardiovascular disease; HTN: hypertension; CKD: chronic kidney disease

These findings directly apply to CDI, age-specific risk adjustment, and informatics tool design. From a CDI perspective, the age-stratified view supports more tailored interventions, such as deploying real-time alerts that consider age and clinical indicators to flag under-documented diagnoses (e.g., prompting liver function review in elderly patients with SIRS). From a quality and reimbursement lens, failure to accurately capture age-associated comorbidities in older adults can lead to undervalued severity of illness (SOI), underestimated risk of mortality (ROM), and loss in case mix index (CMI). Furthermore, this analysis can inform the development of age-sensitive documentation checklists, automated query triggers, or EHR-integrated clinical decision support (CDS) tools to ensure that documentation practices scale appropriately with the clinical complexity of older patients. Ultimately, Figure 4 underscores that while documentation intensifies with age, variability remains in what gets documented, highlighting the need for structured, data-driven documentation strategies that reflect actual clinical burden across all age groups.

## Discussion

The observed documentation rates were substantially lower than expected for most of the comorbidities examined in this study. Hypertension was documented in only 36.6% of patients compared to the expected prevalence of 78% (gap score 0.53, p < 0.001). Diabetes was recorded in 18.3% of patients versus the expected 53% (gap score 0.65, p < 0.001). Anemia was documented in 28% of SIRS patients, while the expected prevalence was 0.66% (gap score 0.58, p < 0.001). Of note, liver failure had the most profound gap with a documentation score of 6.1%, with the expected rate being 57% (gap score 0.89, p < 0.001). CKD and cardiovascular disease also exhibited moderate but significant gaps with observed prevalences of 9.9% (gap score 0.46, p = 0.053) and 20.7% (gap score 0.39, p = 0.013), respectively, versus their expected prevalences of 18% and 34%, respectively.

Figure 1 illustrates a STROBE-compliant flowchart demonstrating the cohort selection process to ensure methodological transparency. The figure depicts the narrowing process from the initial record pull to the final inclusion of 82 patients with confirmed SIRS and organ dysfunction. Table 3 presents the expected versus observed documentation prevalence for each comorbidity, highlighting significant documentation gaps, particularly for liver failure, diabetes, and HTN, with corresponding gap scores. Table 3 also shows the statistical significance of each comorbidity gap using p-values derived from one-sample z-tests. Liver failure, HTN, and diabetes all had significance at p < 0.001, inducing significant underdocumentation rates. Figure 2 visualizes the relationship between benchmarked (expected) and actual (observed) documentation rates of key comorbidities in the SIRS cohort. Figure 3 displays calculated gap scores by comorbidity, categorizing liver failure, anemia, and diabetes as “high-gap” diagnoses, which should be prioritized in CDI interventions. 

The highlighted documentation gaps, especially for liver failure, DM, and HTN, are clinically meaningful, as they can substantially influence patient outcomes, hospital risk adjustment, and reimbursement. Failure to adequately document these diagnoses may lead to the underestimation of patient complexity, adversely affecting clinical decision-making and resource allocation. The findings presented in this study underscore a persistent and clinically consequential gap in the documentation of key comorbidities among patients hospitalized with SIRS. These gaps, most pronounced in liver failure and diabetes, reflect missed opportunities for optimized patient care and may jeopardize hospital performance under national quality measurement frameworks such as the CMS Hospital Star Ratings and the Leapfrog Hospital Safety Grade. In previous work, Liu et al. demonstrated that underreporting chronic comorbidities in sepsis cohorts reduced predictive accuracy of early warning systems and increased ICU admission delays due to incomplete clinical profiles [38]. Further, Schwarzkopf et al. found that sepsis and its risk factors are consistently under-coded in administrative data. This skews hospital performance measures and epidemiologic estimates due to high institution variability [39]. Within today’s healthcare landscape, transparency, accountability, and reimbursement are increasingly tied to quality outcomes; thus, comprehensive clinical documentation is an institutional imperative. The CMS Hospital Star Rating system is structured around five core domains: mortality, safety of care, readmission, patient experience, and timely and effective care. In these domains, risk adjustment methodologies are utilized to ensure hospitals are evaluated justly based on the clinical complexity of their patient populations. As seen in Table 3, the observed prevalence of key chronic conditions fell far below the expected values derived from large-scale epidemiologic and clinical cohort data. For instance, HTN, with a benchmark prevalence of 78% in similar cohorts, was only documented in 37% of this study’s patient sample (Gap Score = 0.53, p < 0.001). Pan et al. highlighted that ICD-10 coding sensitivity for comorbidities has declined over two decades, with some conditions being under-coded in up to 60% of cases, which leads to diminished accuracy in models predicting in-hospital mortality [40]. This underdocumentation results in suppressed HCC weights, leading to falsely lowered severity-adjusted benchmarks for mortality, LOS, and readmission outcomes. The consequence of this data is a distorted view of hospital performance that may negatively influence public ratings and payer perception. 

Similarly, the Leapfrog Safety Grade relies on both process and outcome measures, such as rates of infection, early deterioration detection, and timely clinical response. Patient comorbidities' presence, recognition, and management directly influence all these metrics. For example, early intervention bundles for malnourished or frail patients may not be activated if these comorbidities are not documented at the time of admission. As reported in the data analysis, the under-recognition of anemia (expected: 66%; observed: 28%) and cardiovascular disease (expected: 34%; observed: 21%) suggests that risk stratification tools and early warning systems relying on complete comorbidity profiles may be rendered less effective in this setting. Figure 3 and Table 2 illustrate these disparities, highlighting absolute prevalence gaps and confidence interval-based uncertainty in documentation performance. Similar studies have demonstrated that physician-led documentation improvement initiatives in surgical inpatient groups have resulted in marked increases in case mix index and hashed out contribution margins, confirming that improved comorbidity capture leads to higher reimbursement and increased accuracy for institutional benchmarking [41].

Hospitals that do not accurately capture comorbidity data risk missing opportunities for early clinical intervention and losing traction in quality-based reimbursement programs and competitive benchmarks. Of importance, the CMS Value-Based Purchasing (VBP) program and Hospital Readmission Reduction Program (HRRP) apply financial penalties and rewards based on similar metrics. If documentation does not accurately reflect patient complexity, institutions are exposed to unjustified penalties for what appear to be excessive mortality or additional readmission rates. Studies investigating otolaryngology and vascular surgery cohorts have demonstrated that failure to document comorbidities degraded hospital benchmarks, including publicly reported mortality indices, emphasizing the real-world impacts of incomplete documentation on institutional quality grade cards [42,43]. Table 3 shows the p-values for comorbidity underdocumentation as significant across nearly all conditions, reinforcing the non-random nature of this critical phenomenon, of which the implications extend beyond scoring. Figure 3 demonstrates the plot of comorbidity gap scores for liver failure, anemia, and diabetes as high-gap conditions. These comorbidities are also major clinical contributors to patient deterioration, LOS, and the cost of healthcare. Integrating these findings into hospital documentation improvement strategies is essential. For instance, machine learning-enhanced clinical decision support tools may trigger prompts when high-risk profiles are presented without the corresponding ICD-10 codes. The utilization of this kind of tool integration aligns with the CMS mandate for digital quality reporting that supports the integration of predictive analytics tools across institutions. These discrepancies may reflect systemic variation due to provider documentation behavior or the differential usage of templated notes in the EPIC software. Identifying these documentation gaps is the first step toward targeted provider education, standardization of admission documentation templates, and CDI-focused interventions tailored to important patient demographics. Figure 1 demonstrates a STROBE-compliant overview of the study population to support reproducibility and transparency of this data. Taken together, the results of this study add to the growing body of literature that emphasizes the importance of real-time and structured documentation of key patient comorbidities. The comorbidity capture gap analysis model presented in this study offers a practical and data-driven method for institutions to quantify and close critical documentation gaps. When applied proactively, this kind of approach will not only improve clinical care but also enhance institutional performance in federal quality programs. In an environment where hospital sustainability and public trust rely on perceived and actual healthcare quality, optimizing documentation practices is increasingly becoming a strategic priority with measurable downstream benefits.

Stratification analysis by age and sex

Chi-square analyses stratified by age group showed statistically significant variation in HTN documentation (χ² = 10.92, p = 0.012), with older patients (51-70 years) more likely to have documentation of HTN. CKD (p = 0.101), DM (p = 0.197), anemia (p = 0.14), liver failure (p = 0.501), CHF (p = 0.10), and mortality status (p = 0.57) did not show statistically significant differences across age groups.

Sex-stratified analysis showed a significant association between CKD documentation and sex (χ² = 3.90, p = 0.048), with male patients demonstrating higher documentation rates. However, DM (p = 0.375), HTN (p = 0.67), anemia (p =0.284), liver failure (p = 0.603), CHF (p = 0.281), and mortality status (p = 0.851) did not exhibit significant sex-based differences in documentation.

Association with mortality

Through examining inpatient mortality, the chi-square test did not show any significant associations between documented comorbidities and mortality rates (χ² = 0.02, p = 0.89). This suggests that underdocumentation may reflect practices in systemic documentation, rather than practices in patient acuity or clinical outcomes.

Limitations

There are several limitations regarding this study that should be noted. Firstly, the data utilized were derived from a single academic medical center, which may limit the generalizability of these findings regarding their application to other institutions with different documentation practices, patient populations, or EHR systems. Resultingly, the observed rates of comorbidity underdocumentation highlighted may not be representative of broader healthcare settings and institutions. The comorbidity identification process also relied on manual chart abstraction and review, which, despite careful review, is inherently subject to human error. Efforts were made to standardize the abstraction process; however, variation in clinical note structure, technology usage, and conflicting documentation may have introduced inconsistencies within each patient's chart. While the study benchmarks observed documentation rates against established prevalence estimates gathered from literature, these external benchmarks may not wholly reflect the true expected prevalence within the specific clinical context of each unique SIRS patient. Disease burden and comorbidity patterns can substantially vary depending on patient acuity and case. General prevalence data may underestimate or overestimate expected rates in critically ill cohorts. Further, this analysis did not adjust for comorbidity severity, duration, or clinical relevance during the index admission. As such, it is possible that some conditions, although present, were not documented due to clinical inactivity or lack of relevance to the primary diagnosis at the time of patient admission. This limitation highlights the need to interpret underdocumentation not just as an omission, but within the clinical relevance of the episode of hospitalization. Lastly, this study did not incorporate potentially influential factors relating to the status of ICU admission, LOS, or socioeconomic distinctions, all of which may affect documentation practices and outcomes. These variables were either unavailable in the EHR or inconsistently documented in the retrospective data set. These limitations represent areas for future prospective investigations.

Future implications

The results of this study include significant implications regarding the design and utilization of next-generation clinical documentation support systems, especially those using real-time informatics or artificial intelligence (AI) but lack statistical power. By quantifying the gap between clinically expected and observed documentation of high-impact comorbidities in patients with SIRS and acute organ dysfunction, this study can provide an objective framework with the potential to be translated into an actionable framework for use within AI-augmented clinical documentation improvement (AI-CDI) platforms. AI-CDI systems utilize machine learning and natural language processing to analyze both structured and unstructured EHR data to enable the automatic identification of documentation gaps and generate context-sensitive prompts for clinicians in real-time.

This framework addresses a critical unmet need in current documentation workflows: the difficulty of dynamically identifying documentation omissions that have clinical and financial consequences.

This comorbidity capture gap model can be scalable and converted into an algorithm with real-time surveillance of structured data fields in the EHR. For instance, renal replacement therapy orders, consistently elevated creatinine values, or nephrology consults without an associated ICD-10-CM diagnosis for CKD can be automatically flagged for a documentation opportunity in the patient’s chart. These critical intelligent alerts can also be delivered in a multitude of ways, such as passively through EPIC’s SlicerDicer or Clarity-based dashboards, or actively through Best Practice Advisories (BPAs), Epic Smart Suggestions, or third-party CDI plug-ins with HL7/FHIR integration. These tools would allow for real-time prompting of clinicians, CDI specialists, or coding teams during patient care or even following documentation review.

The broader implication of this study is regarding shifting from retrospective and manual documentation audits towards proactive and informatics-driven CDI interventions. By embedding comorbidity capture scores and risk-adjustment benchmarks directly into EHR, health systems can efficiently enhance their ability to improve accurate and complete patient documentation, increase HCC and DRG alignment for financial optimization, and ensure patient severity of illness is accurately represented in institutional quality metrics. Further, this methodology can complement and strengthen existing clinical decision support (CDS) infrastructure, especially concerning sepsis early warning systems, by strengthening documentation completeness with patient acuity and care prioritization.

From the standpoint of policy and administration, integration of real-time bioinformatics tools aligns with the CMS goal to move towards value-based care and risk-adjustment payment models. Incorporating logic from AI-CDI derived from studies such as this can support compliance with endorsed measures from the National Quality Forum (NQF) and improve healthcare transparency and accuracy in publicly reported patient outcomes. Future research may extend this model to other high-risk patient diagnostic categories to evaluate the impact of real-time CDI alerts on coding accuracy and chart completeness. Ultimately, this study aims to underscore the importance of aligning clinical practice with documentation integrity through the continuous development and utilization of intelligent EHR-integrated systems that reflect and enhance the complexity of patient care. 

Bias considerations

It is important to consider several points of consideration regarding this study’s possible subject to several potential sources of bias, notably those inherent in retrospective EHR-based analyses. Selection bias may have been introduced by relying only on patients with the ICD-10-CM code R65.11 for “Systemic inflammatory response syndrome [SIRS] of non-infectious origin with acute organ dysfunction”, which may not uniformly capture all patients who clinically met criteria for SIRS, especially patients whose conditions evolved or resolved earlier during their admission course. Chart abstraction was also dependent on available documentation within patient medical records, meaning that the absence of a recorded comorbidity was interpreted as non-documentation rather than true absence of the comorbidity of interest, thus introducing the risk of information bias. This bias is particularly relevant in acute care settings, where high provider workload, variable practices in documentation style, and the prioritization of immediate clinical concerns may contribute to an incomplete problem list or the omission of certain chronic comorbidities from the patient’s official record. Provider documentation habits and styles, familiarity with coding requirements, and even varying usage of templated notes may significantly differ from provider to provider, potentially influencing whether a given diagnosis was included in the EHR. These biases can potentially lead to the systemic underestimation of comorbidity prevalence and capture, affecting the accuracy of calculated documentation gaps. While every effort was made in this study to ensure data consistency and completeness, these limitations are inherent to real-world EHR data and should be acknowledged when interpreting these findings.

## Conclusions

The large gaps in documentation, particularly for liver failure and diabetes, indicate areas where clinical documentation efforts should be prioritized. Accurate and complete documentation is also vital for appropriate case-mix adjustment, reimbursement accuracy, and guiding real-time care escalation. For example, unrecognized or underdocumented liver failure may delay interventions critical for monitoring and patient care, given its known association with adverse outcomes in critically ill and SIRS patients. The findings of this study corroborate prior studies demonstrating the chronic underrecognition and underdocumentation of comorbidities in acute care settings. The notably high gap score of 0.89 for liver failure far exceeds expectations in SIRS patient cohorts, demonstrating a need for enhanced clinical awareness and systemic documentation improvements that can improve patient outcomes and care. Underdocumentation of the key comorbidities highlighted in this study may overshadow chronic disease recognition in clinical workflows.
